# Dynamic Control of Synchronous Activity in Networks of Spiking Neurons

**DOI:** 10.1371/journal.pone.0161488

**Published:** 2016-09-26

**Authors:** Axel Hutt, Andreas Mierau, Jérémie Lefebvre

**Affiliations:** 1 Deutscher Wetterdienst, Section FE12 - Data Assimilation, 63067, Offenbach am Main, Germany; 2 Institute of Movement and Neurosciences, German Sport University, Cologne, Germany; 3 Krembil Research Institute, University Health Network, Toronto, Ontario, M5T 2S8, Canada; 4 Department of Mathematics, University of Toronto, Toronto, Ontario, M5S 3G3, Canada; McGill University Department of Physiology, CANADA

## Abstract

Oscillatory brain activity is believed to play a central role in neural coding. Accumulating evidence shows that features of these oscillations are highly dynamic: power, frequency and phase fluctuate alongside changes in behavior and task demands. The role and mechanism supporting this variability is however poorly understood. We here analyze a network of recurrently connected spiking neurons with time delay displaying stable synchronous dynamics. Using mean-field and stability analyses, we investigate the influence of dynamic inputs on the frequency of firing rate oscillations. We show that afferent noise, mimicking inputs to the neurons, causes smoothing of the system’s response function, displacing equilibria and altering the stability of oscillatory states. Our analysis further shows that these noise-induced changes cause a shift of the peak frequency of synchronous oscillations that scales with input intensity, leading the network towards critical states. We lastly discuss the extension of these principles to periodic stimulation, in which externally applied driving signals can trigger analogous phenomena. Our results reveal one possible mechanism involved in shaping oscillatory activity in the brain and associated control principles.

## Introduction

Brain signals are rife with oscillatory spectral patterns. These rhythmic features, uncovered through both intracranial and non-invasive recordings, have been shown to correlate strongly with cognitive processes, memory, and sensorimotor behavior [1, 2, 3], and are thus believed to be dynamic signatures of specific neural computations. As such, oscillatory activity is ubiquitous throughout the nervous system and represents the focus of an effervescent area of research [4, 5, 6].

Brain oscillations are however far from being static. Indeed, cortical rhythms are commonly subjected to sudden shifts induced by variations in behavior and cognitive states. Such spectral transitions are notably observed across normal sleep stages [7] or during the recruitment of attention [8]. The variability of oscillatory neural activity within the gamma band has been thoroughly studied and linked to changes in visual stimuli statistics and timely adjustments in local synaptic wiring [9, 10, 11]. However, shifts in brain oscillatory activity are also reliably observed at slower frequencies. Alpha oscillatory activity, in particular, has been found to be highly volatile [12]. It has been found that the iAPF accelerates during cognitive [13], memory [14] and sensorimotor [15] task performance as well as following a strenuous bout of physical exercise [16]. In addition, recent studies provide strong evidence that alpha oscillations are one candidate mechanism for gating the temporal window of sensory integration, and thus dictating the resolution of conscious sensory updating. Specifically, deliberate alterations of the iAPF within individual subjects, induced by transcranial alternating current stimulation, has been found to influence visual stimuli [17]. Consistent with this, individuals with higher iAPF have vision with finer temporal resolution, and within an individual, spontaneous fluctuations in iAPF predict visual perception [18]. Furthermore, in a temporal cueing task, forming predictions about when a stimulus will appear can instantaneously bias the phase of ongoing alpha-band oscillations toward an optimal phase for stimulus discrimination [19]. Taken together, these findings suggest that the magnitude of the iAPF is indicative of the level of arousal/attention, preparedness and performance of recruited cortical nets, in which “the faster the better”. Given that alpha activity operates on much broader spatial and temporal scales [20], spectral transitions observed within those frequency ranges likely relies on more global and distributed mechanisms.

To this day, the mechanism supporting these larger scale frequency transitions has been poorly understood. A key question is whether such transitions could be triggered by external stimulation. Recent studies have indeed shown that weak electric fields can perturb individual alpha oscillations and have a direct effect on visual stimulus perception [17,21] and task performance by reinforcing endogenous slow-wave rhythms [1,22].

To explore this question, we here investigate a non-linear network of spiking neurons with time delay, exhibiting alpha-like oscillatory activity. Our results reveal that despite the noisiness of the connectivity, stimuli implement an online gain-control mechanism where the peak frequency reflects the activation state of the neurons. We show that neural inputs, here modeled by noise, change the shape of the neuron response function, significantly changing the systems equilibria and stability. Using mean-field analysis of the network collective dynamics, we demonstrate that noise causes the system to shift from slow non-linear oscillations to fast linear oscillations, bringing the system towards a critical state. We also derive a frequency tuning curve that relates the network’s synchronous frequency to the noise intensity driving its constituent neurons. We lastly explore how these results also apply to periodic stimuli, opening new perspectives on how external brain stimulation can be used to control neural synchronous activity.

## Model

**Fig 1:**
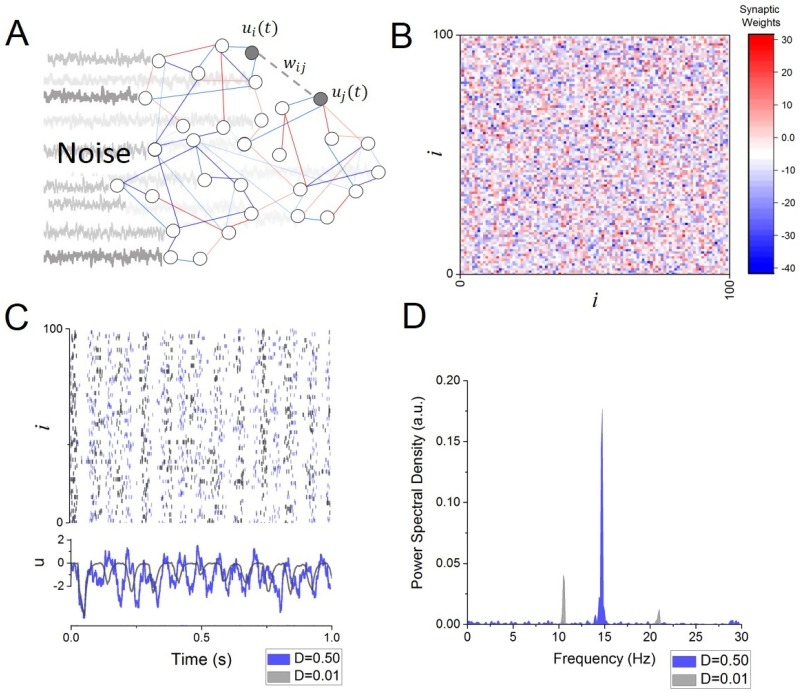
Frequency transitions in a random network of spiking neurons. A. Schematic illustration depicting some features of the network model, in which interconnected cells are driven by independent sources of noise. Individual cells are connected via excitatory (red) and inhibitory (blue) synaptic connections. B. Synaptic connectivity matrix. Weights are randomly distributed around a mean value *g* (See Eq 2). C. Sample network activity, in which neurons spike timing is modulated by global, slow-wave synchronous oscillations in both low (grey; D = 0.01) and high (blue; D = 0.50) input conditions. Faster and more irregular firing modulations characterize the high-input state. D. Power spectral density of the network mean activity $\bar{u}$ in low (grey; D = 0.01) and high (blue; D = 0.50) input conditions. Other parameters are *α* = 100Hz, *β* = 300mV, *g* = −10mV/Hz, *s* = 20mV/Hz, *τ* = 25ms.

In the present work, we analyze the dynamics of a generic network of spiking neurons (Fig 1A) whose membrane potential *u*_*i*_(*t*) evolves according to the following set of non-linear differential equations

$$\alpha^{-1}\frac{d}{dt}u_{i}\left(t\right)=-u_{i}\left(t\right)+N^{-1}\Sigma_{j=1}^{N}w_{ij}X_{j}\left(t-\tau \right)+I_{i}\left(t\right)$$ (1)

where *α* is the membrane time constant, *w*_*ij*_ = [***W***]_*ij*_ are synaptic weights and where *τ* is a mean conduction delay. The membrane potential *u*_*i*_(*t*) represents the deviation from the neurons resting potential that is present in the absence of synaptic and external input. The presynaptic spike trains $X_{j}\left(t\right)=\Sigma_{\left\{t_{l}\right\}}\delta \left(t-t_{l}\right)$ obey the non-homogenous Poisson processes *X*_*i*_ → Poisson(*f*[*u*_*i*_]) with rate *f* and the Dirac distribution *δ*(*t*). The firing rate function *f* has a non-linear sigmoid shape and is defined by *f*[*u*_*i*_] = (1 + exp[−*βu*_*i*_])^−1^,i.e. the maximum firing rate approaches *f* = 1 for large membrane potentials. All neurons are subjected to afferent pre-synaptic inputs. The synaptic connectivity scheme was randomly set (Fig 1B), such that

$$w_{ij}=g+s\;\eta_{ij},$$ (2)

where *g* is the mean synaptic strength, *s* is the weight variance and *n*_*ij*_ are zero-mean independent Gaussian white noise such that < *n*_*ij*_*n*_*kl*_ >_*NxN*_ = *δ*_*ik*_*δ*_*jl*_ with the Kronecker symbol *δ*_*nm*_ and where < >_*NxN*_ is an average evaluated over all possible pairs of indices of the matrix ***W***. We also consider other sources of synaptic inputs which we model as stochastic elements $I_{i}\left(t\right)=\sqrt{2D}\xi_{i}\left(t\right)$ given the independent Gaussian white noise processes *ξ*_*i*_ with zero mean and < *ξ*_*i*_*ξ*_*j*_ >_*T*_ = *δ*_*ij*_, where < >_*T*_ is an average evaluated over an epoch of duration *T* or over an ensemble of realizations of processes. Such noise is meant to represent the effect of synaptic bombardment on neural membrane potentials. Such inputs can be shown to be well approximated by Gaussian processes in the diffusion limit case [23,24] and this is the approach we use in the following analysis. In the present work, we also consider a network of neurons whose spatial mean synaptic action is inhibitory with < 0.

Under the choice of a specific synaptic connectivity and other model parameters, the network spontaneously engages in synchronous activity at a baseline frequency of 10Hz while external input is absent. However, this frequency is found to be highly volatile in the presence of stochastic input: if the noise level is increased, the network spiking activity becomes more irregular and the synchronous frequency increases, cf. Fig 1C. The power spectrum of the network mean activity is plotted in Fig 1D, where peaks can be observed at the systems natural frequency and higher harmonics. As noise intensity is increased in the system, the peak frequency and associated harmonics gradually shift towards higher a frequency range.

## Mean Field Representation for Stochastic Input

To better understand the impact of inputs on the dynamics, let us derive mean-field equations for our system. In the limit where the number of neurons is large, i.e. *N* → *∞*, and the mean firing time scale 1/*f* is much smaller than the time scale of dendritic currents [25] we can use the response function *f* to approximate the rate at which recurrent pre-synaptic inputs perturb the activity of a neuron. To this end, one averages *u*_*i*_(*t*) over a very short time window and introduces a so-called coarsening in time [25, 26]. This well-established transformation allows to translate population spiking activity to population rate dynamics

$$N^{-1}\Sigma_{j=1}^{N}w_{ij}X_{j}\left(t-\tau \right)\approx N^{-1}\Sigma_{j=1}^{N}w_{ij}f\left[u_{j}\left(t-\tau \right)\right]$$ (3)

In the following, we use the same symbol for the original and the temporally coarse-grained membrane potential for notational simplicity. Let us further assume that emerging oscillations occur in a mean-driven regime in which the local dynamics can be seen as small independent fluctuations around the network mean activity i.e.

$$u_{i}\left(t\right)=\bar{u}\left(t\right)+\nu_{i}\left(t\right)$$ (4)

where the network mean activity is given by

$$\bar{u}\left(t\right)=N^{-1}\;\Sigma_{i=1}^{N}\;u_{i}\left(t\right)\equiv <u{>}_{N}$$ (5)

and < >_*N*_ is an average performed over the *N* unit of the network. In the following, we re-scale time by *αt* → *t* for notational simplicity. As an ansatz, local fluctuations *v*_*i*_ from the mean obey the Ornstein-Uhlenbeck processes

$$\frac{d}{dt}v_{i}=-v_{i}+\sqrt{2D}\xi_{i}\left(t\right).$$ (6)

Then, using Eq (4) above, and taking the mean over *N* neurons

$$\frac{d}{dt}\bar{u}\left(t\right)=-\bar{u}\left(t\right)+<N^{-1}\;\Sigma_{i=1}^{N}\;w_{ij}f\left[\bar{u}\left(t-\tau \right)+v_{j}\left(t-\tau \right)\right]{>}_{N}$$ (7)

Then, as *N* → *∞* [27],

$$<N^{-1}\;\Sigma_{i=1}^{N}w_{ij}f\left[\bar{u}\left(t-\tau \right)+v_{j}\left(t-\tau \right)\right]{>}_{N}\;\approx \;\bar{w}\;\int_{-\infty }^{+\infty }f\left(\bar{u}\left(t-\tau \right)+v\right)\rho \left(v\right)dv$$ (8)

where *ρ* is the probability density function of the solution of Eq (6), i.e. a zero-mean Gaussian distribution with variance *var*[*v*] = *D*. Moreover $\bar{w}\;=\;<w_{ij}{>}_{N}=g$ is the mean network connectivity. In Eq (8), we have used the fact *E*[*XY*] = *E*[*X*]*E*[*Y*] for the expectation value E of a product of two statistically independent random variables X and Y. This applies in Eq (8) since *v*_*j*_ and *w*_*ij*_ are statistically independent.

Whenever the response function gain *β* is very large, [*u*] ≈ *H*[*u*], where *H* is the Heaviside step function with *H*[*u*] = 0 for all *u* < 0 and *H*[*u*] = 1,*u* ≥ 0. Then the right hand side of Eq (8) reads

$$\bar{w}\int_{-\infty }^{+\infty }H\left(\bar{u}\left(t-\tau \right)+v\right)\rho \left(v\right)dv\approx \frac{\bar{w}}{2}\left(1+\text{erf}\left[\frac{\bar{u}\left(t-\tau \right)}{\sqrt{2D}}\right]\right).$$ (9)

Combining previous results, the mean-field dynamics of our spiking network can be well approximated by the scalar non-linear delay-differential equation

$$\frac{d}{dt}\bar{u}\left(t\right)=-\bar{u}\left(t\right)+\frac{\bar{w}}{2}\left(1+\text{erf}\left[\frac{\bar{u}\left(t-\tau \right)}{\sqrt{2D}}\right]\right)$$ (10)

**Fig 2:**
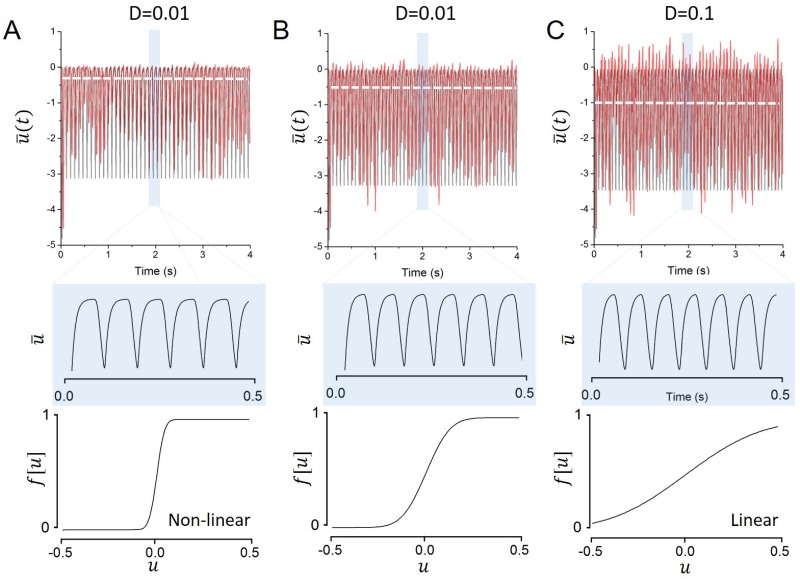
As noise increases, global oscillations accelerate and become gradually more linear. Pre-synaptic noise generically results in a linearization of the neurons response function, altering the network stability and further shaping the frequency of ongoing oscillations. The network mean activity $\bar{u}$ is shown (top panel) with a close up view of a few cycles (middle panel) with the associated response function (bottom panel), for various levels of noise. A. *D* = 0.001. B. *D* = 0.01. C. *D* = 0.1. Other parameters are identical to parameters used in Fig 1.

Eq (10) shows that pre-synaptic noise generically results in a linearization of the neurons response function [28,29] cf. Fig 2, bottom panels. This linearization shapes the input-output relationship of driven neurons, but also alters the features displayed by emergent activity patterns such as synchronous oscillations [30]. Fig 2 illustrates that increasing the external noise tunes the frequency of the network mean activity.

## Stochastic Stability Analysis

**Fig 3:**
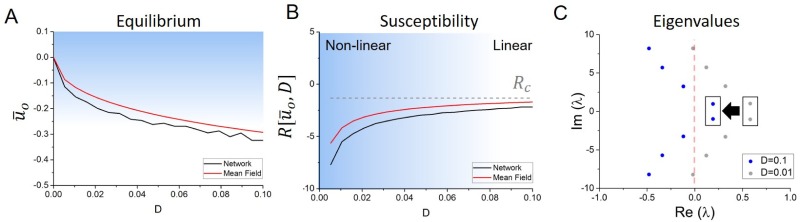
Network stability and equilibrium are shaped by noise. A. Fixed point of the system as per Eq (11) as a function of increasing noise intensity. Noise generically decreases the equilibrium, due to an increased recruitment of recurrent connections. B. Network susceptibility as a function of noise intensity. A gradual shift towards the critical susceptibility *R*_*c*_ occurs under the action of noise, causing the system to transit from slow non-linear oscillations to fast linear oscillations. C. System’s eigenvalues for moderate (D = 0.01) and strong (D = 0.1) noise levels. The eigenvalues gradually shift towards the left hand side of the imaginary plane. Critical eigenvalues (pairs of roots inside the black boxes) translate towards the imaginary axis (Re(*λ*) = 0) i.e. closer to the critical state. Other parameters are *α* = 100Hz, *β* = 2500/mV, *g* = −2mV/Hz, *s* = 4mV/Hz, *τ* = 25ms.

To investigate the frequency tuning observed in Fig 2, we take a closer look at the solutions of Eq (10). Synchronous activity in our network emerges as a consequence of an expected supercritical Hopf bifurcation commonly seen in recurrent delayed nets [31]. The single fixed point ${\bar{u}}_{o}$ of $\bar{u}$ result from Eq (10) by setting $\frac{d\bar{u}}{dt}=0$

$${\bar{u}}_{o}=\frac{\bar{w}}{2}\left(1+\text{erf}\left[\frac{{\bar{u}}_{o}}{\sqrt{2D}}\right]\right)\approx -\frac{\sqrt{\pi D}g}{\sqrt{2}g-2\sqrt{\pi D}},$$ (11)

where the last equation assumes small noise intensity *D*. Fig 3A shows that the mean-field fixed point ${\bar{u}}_{o}$ decreases with increasing noise level.

We use the knowledge about the noise-dependent fixed point ${\bar{u}}_{o}$ to better understand the network stability. The linearization of the mean-field Eq (10) about the fixed point ${\bar{u}}_{o}$ from Eq (11) yields

$$\frac{d}{dt}w\left(t\right)=-w\left(t\right)+R\left[{\bar{u}}_{o},D\right]w\left(t-\tau \right)$$ (12)

with deviations *w* from the fixed point where $R\left[{\bar{u}}_{o},D\right]=\frac{\bar{w}}{\sqrt{2\pi D}}\text{exp}\left[-\frac{{\bar{u}}_{o}^{2}}{2D}\right]$ is the network susceptibility. Stability of the network equilibrium ${\bar{u}}_{o}$ is determined by setting $w\left(t\right)=\tilde{u}e^{\lambda t}$ with λ∈ ℂ leading to the characteristic equation

$$\lambda =-1+R\left[{\bar{u}}_{o},D\right]e^{-\lambda \tau }.$$ (13)

Solutions to Eq (13) define the spectrum of the linearized system in Eq (12). Collective oscillatory solutions form in the network if the susceptibility reaches a critical value *R*_*c*_ for *λ* = *i ω*_*c*_ satisfying the complex relationship

$$i\omega_{c}=-1+R_{c}e^{-i\;\omega_{c}\tau }$$ (14)

where $\omega_{c}=\sqrt{R_{c}-1}$ is the critical Hopf frequency i.e. the frequency of network synchronous oscillations close to the instability.

As seen in Fig 3B, in the absence of noise, the susceptibility is well below the critical value, and the network exhibits strong non-linear oscillations. This implies that *R* < *R*_*c*_ for *D* = 0 and the system evolves in a nonlinear limit cycle oscillation beyond a supercritical Hopf bifurcation while the fixed point is asymptotically unstable. As such, in the weak noise limit, the system is set robustly in the synchronous state.

However, upon the presence of noise, linearization of the neurons response function translates into a gradual increase of the susceptibility towards *R*_*c*_: global synchronous oscillations in the network not only accelerate under the action of noise, but also becomes more linear. As such, noise brings the system closer to the bifurcation threshold and hence moves the network towards a critical asynchronous state. The effect of noise on the characteristic eigenvalue spectrum is plotted in Fig 3C. As noise intensity increases, the eigenvalues undergo a gradual shift towards the left hand side of the complex plane, bringing pairs of eigenvalues closer to the imaginary axis and thus closer to the bifurcation threshold. This occurs because the system’s susceptibility approaches the critical susceptibility under the effect of noise. As such, afferent inputs engage the network and drive it towards the asynchronous state and the transition trajectory in parameter space is characterized by an gradual increase in the network peak frequency.

## Stochastic Frequency Tuning

Finding explicit frequency relationships in the fully non-linear regime is challenging. Yet, to approximate the dependence of the network frequency on the noise driving the neurons, one might use the Galerkin method [32, 33]. Whenever *f*[*u*] ≈ *H*[*u*] holds for sufficiently large values of the gain β, this approach seeks to find a frequency *ω* minimizing the measure

$$J=\int_{0}^{2\pi /\omega }\left(\frac{d}{dt}\bar{u}-F\left[\bar{u}\right]\right)\cdot \text{cos}\left(\omega t\right)dt$$ (15)

where $F\left[\bar{u}\right]=-\bar{u}\left(t\right)+\frac{\bar{w}}{2}\left(1+\text{erf}\left[\frac{\bar{u}\left(t-\tau \right)}{\sqrt{2D}}\right]\right)$. Using the ansatz $\bar{u}\left(t\right)=A\text{cos}\left(\omega t\right)+{\bar{u}}_{o}$ while expanding *F* to third order about the steady state ${\bar{u}}_{o}$, one obtains for the first iteration,

$$J=-\frac{1}{2}\frac{A\left(\sqrt{2}\;\pi \;\bar{w}\text{cos}\left(\omega \tau \right)-2{\sqrt{\pi }}^{3}\sqrt{D}\right)}{\omega \sqrt{\pi D}}.$$ (16)

For *J* = 0, one can solve Eq (16) for *ω* > 0 to obtain a first order approximation for the stochastic frequency

$$\omega \approx \frac{1}{\tau }\;{\text{cos}}^{-1}\left(\frac{\sqrt{2\pi D}}{\bar{w}}\right)\;.$$ (17)

**Fig 4:**
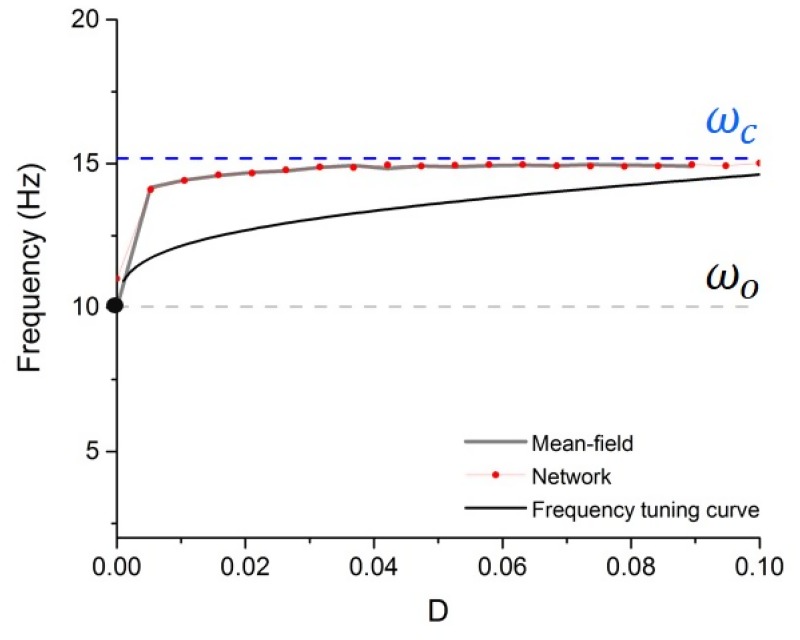
Frequency tuning curve. Frequency of the network synchronous oscillations as a function of noise intensity. Noise causes the peak frequency of the network oscillations to shift from the baseline frequency *ω*_*o*_ towards the critical frequency *ω*_*c*_. The peak frequency is plotted according to numerical simulations of the network dynamics (red dotted curve), the mean-field approximation (grey; as per Eq 10) and using the frequency tuning curve (black; as per Eq 17). Other parameters are taken from Fig 3.

Expanding to third order for weak noise, i.e. *D* ≈ 0

$$\omega \approx \frac{\pi }{2\tau }-\frac{\sqrt{2\pi D}}{\bar{w}\tau }-\frac{1}{3}\frac{\sqrt{2\pi^{3}D^{3}}}{{\bar{w}}^{3}\tau }+O\left(D^{5/2}\right)$$ (18)

leading to the frequency tuning curve

$$\omega \approx \omega_{o}\cdot \left(1+\Delta \left(D\right)\right)$$ (19)

where Δ(*D*) is a noise-induced shift with Δ(*D*) > 0. Fig 4 shows the frequency with respect to the noise intensity. Together with the results above, while approximate, demonstrate that under the action of noise, the network oscillation frequency gradually shifts from a non-linear baseline frequency $\omega_{o}=\frac{\pi }{2\tau }\approx \;10\text{Hz }(D=0)$ towards the critical frequency $\omega_{c}=\sqrt{R_{c}-1}\approx 15\text{Hz}$. Eqs (18) and (19) thus imply that as noise increases in the system, the network transits from slow non-linear rhythms to fast linear oscillations. This can also be understood by looking at the susceptibility, which gauges the relative influence of recurrent interactions in the network, plotted in Fig 3B. As susceptibility decreases under the action of noise, the system accelerates and shifts from a recurrent deeply synchronous state to an asynchronous input driven regime.

## Mean Field Analysis for Periodic Stimulation

We have seen that noise shapes the stability and oscillatory features of non-linear networks by linearizing the neurons response function. However, this feature is not exclusive to noisy inputs. Indeed, numerous studies have shown that, in addition to changes in brain state, ongoing oscillatory activity can be modulated by noninvasive stimulation, something that is increasingly capitalized upon in basic research and clinical practice [34, 35, 36, 37, 38, 39]. Recent results have further shown that exogenous electric rhythmic stimulation (i.e. periodic forcing), in addition to resonance and entrainment, can also provoke non-linear acceleration of endogenous oscillations and shift the baseline frequency of driven neural systems [40], causing resonance curves and Arnold tongues to bend in stimulation parameter space ([40] cf Fig 4). To explore the mechanism behind this non-linear effect, we here revisit the analysis performed in the stochastic case and adapt it to the presence of periodic forcing.

In the presence of global periodic stimulation with frequency *f*_*s*_ and amplitude *I*_0_, the network dynamics obeys

$$\frac{d}{dt}u_{i}\left(t\right)=-u_{i}\left(t\right)+N^{-1}\;\Sigma_{j=1}^{N}w_{ij}X_{j}\left(t-\tau \right)+\;I_{0}\text{sin}\left(2\pi f_{s}t\right).$$ (20)

First, we consider activity course-grained in time with

$$\Sigma_{j=1}^{N}w_{ij}X_{j}\left(t-\tau \right)\approx \Sigma_{j=1}^{N}w_{ij}f\left[u_{j}\left(t-\tau \right)\right]$$ (21)

similar to the stochastic case. Then we assume two temporal scales in the evolution of the potentials with *u*_*j*_ = *m*_*j*_ + *v*_*j*_: the slow mode *m*_*j*_ and the fast mode *v*_*j*_. Inserting this relation into Eq (20), we obtain

$$\frac{d}{dt}v_{i}=-v_{i}+I_{0}\text{ sin}\left(2\pi f_{s}t\right)$$ (22)

and

$$\frac{d}{dt}m_{i}\left(t\right)=-m_{i}\left(t\right)+N^{-1}\;\Sigma_{j=1}^{N}w_{ij}f\left[m_{j}\left(t-\tau \right)+v_{j}\left(t-\tau \right)\right]$$ (23)

Since the experimental observation reflects the average activity in the neural ensemble, we consider spatially homogeneous slow activity $m_{j}=\bar{u}\left(t\right)$. Then averaging over the time interval of one short stimulus cycle, one can express the spatio-temporal mean dynamics $\bar{u}\left(t\right)$ in the adiabatic regime for fast stimuli

$$\frac{d}{dt}\bar{u}\left(t\right)=-\bar{u}\left(t\right)+<N^{-1}\;\Sigma_{j=1}^{N}w_{ij}f\left[\bar{u}\left(t-\tau \right)+v_{j}\left(t-\tau \right)\right]{>}_{T,N}.$$ (24)

Here <·>_*T*,*N*_ denotes a spatial average and a time average taken over a time interval *T* small compared to the dynamics of the network i.e. for 1/*f*_*s*_ ≤ *T* ≪ 2*π*/*ω*_o_, i.e.

$$<u_{i}\left(t\right){>}_{T,N}=\sum_{i=1}^{N}\int_{t}^{t+1/f_{s}}u_{i}\left(t\right)\;dt$$ (25)

for the local activity *u*_*i*_(*t*). Again, this implies that the driving frequency *f*_*s*_ is large compared to the systems self-sustained oscillation frequency. By virtue of this time scale separation, it is reasonable to assume stationarity in the time interval of duration *T*. The solutions of Eq (22) for large frequencies and large times *t* → *∞* obey

$$v_{i}\left(t\right)\approx -\frac{I_{0}}{2\pi f_{s}}\text{cos}\left(2\pi f_{s}t\right),$$ (26)

i.e. the fluctuations about the spatial mean synchronize and converge to a single solution. Then

$$<N^{-1}\;\Sigma_{j=1}^{N}w_{ij}f\left[\bar{u}\left(t-\tau \right)+v_{j}\left(t-\tau \right)\right]{>}_{T,N}\approx \bar{w}\int_{-\infty }^{+\infty }f\left(\bar{u}\left(t-\tau \right)+v\right)\rho \left(v\right)dv$$ (27)

with the probability density

$$\rho \left(v\right)=\int_{t}^{t+1/f_{s}}\delta \left(v+\frac{I_{0}}{2\pi f_{s}}\text{cos}\left(2\pi f_{s}t\right)\right)dt=\;\frac{1}{\pi \sqrt{\mu^{2}-v^{2}}}$$ (28)

with *μ* = *I*_0_/2*πf*_*s*_. Here, we have used the time scale separation between the slow evolution of $\bar{u}\left(t\right)$ and the fast synchronous fluctuations. Eq (28) has the same form as Eq (8) in the case of stochastic stimulation. Again, similar to the stochastic case, the external stimulation leads to a convolution of the nonlinear response function *f* with the probability density function of the stimulus-induced fluctuations about the spatial mean. We note that, in contrast to the stochastic case, the notion of a probability density function in the present deterministic system may appear counter-intuitive. This interpretation however is reasonable since *ρ*(*v*) is proportional to the residence time of the oscillation *v*_*i*_(*t*) at amplitude *v* [41] that motivates the interpretation of a probability density.

Now assuming [*u*] ≈ *H*[*u*], Eq (28) reads

$$\tilde{F}\left[\bar{u}\right]\approx \int_{-\mu }^{\mu }\frac{f\left(\bar{u}\left(t-\tau \right)+v\right)}{\pi \sqrt{\mu^{2}-v^{2}}}dv=\;\frac{1}{\pi }\;{\text{sin}}^{-1}(\bar{u}/\mu )+\frac{1}{2},\;-1\leq \bar{u}/\mu \leq 1$$ (29)

and $\tilde{F}\left[\bar{u}\right]=1$ for $\frac{\bar{u}}{\mu }>1$ and $\tilde{F}\left[\bar{u}\right]=0$ for $\frac{\bar{u}}{\mu }<-1$. Combining previous results, the dynamics in presence of periodic forcing becomes

$$\frac{d}{dt}\bar{u}\left(t\right)=-\bar{u}\left(t\right)+\bar{w}\tilde{F}\left[\bar{u}\left(t-\tau \right)\right]$$ (30)

## Stability and Frequency Tuning

**Fig 5:**
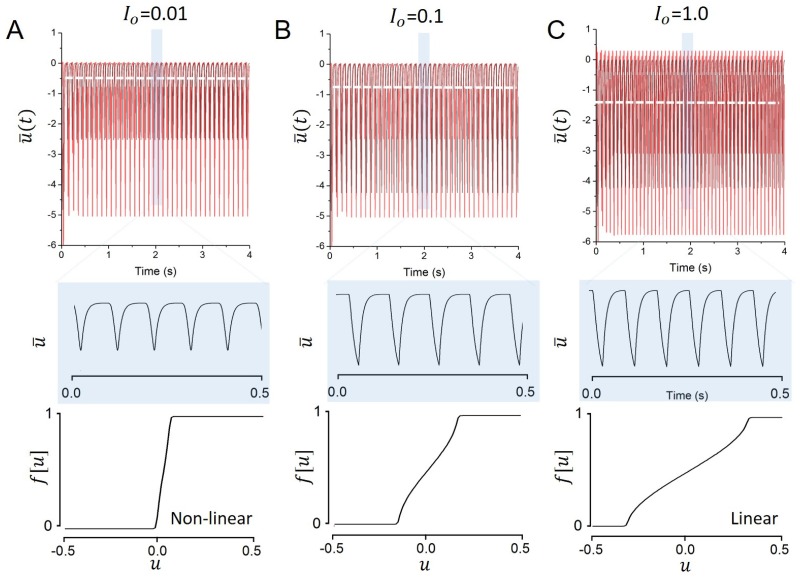
As period driving amplitude increases, global oscillations accelerate and become gradually more linear. The network mean activity $\bar{u}$ is shown (top panels) with a close up view of a few cycles (center panels) with the associated response function (bottom panels), for various input amplitudes. A. *I*_*o*_ = 0.01. B. *I*_*o*_ = 0.1. C. *I*_*o*_ = 1.0. Other parameters are *α* = 100Hz, *β* = 2500/mV, *g* = −2mV/Hz, s = 4mV/Hz, *τ* = 25ms.

Taking a closer look at the slope of the new response function ${\tilde{F}}^{\prime }\left[u\right]=d\tilde{F}/du$, we find

$$\frac{d\tilde{F\prime }}{d\mu }<0$$ (31)

for $-\mu \leq \bar{u}\leq \mu$, and $\frac{d\tilde{F\prime }}{d\mu }=0$ otherwise. Hence the nonlinear response function flattens and becomes increasingly linear for increasing *μ* similar to the stochastic case for increased noise level. Fig 5 (bottom panels) shows $\tilde{F}$ for three different stimulus amplitudes, i.e. different values of μ, confirming this analytical finding.

To gain insight into the dynamics of $\bar{u}$, we consider the fixed point ${\bar{u}}_{o}$ and small deviations about it. Similar to Eq (12), the corresponding characteristic roots are defined by

$$\lambda =-1+R\left[{\bar{u}}_{o},\mu \right]e^{-\lambda \tau }$$

with the susceptibility $R=\bar{w}{\tilde{F}}^{\prime }\left[{\bar{u}}_{o}\right]$. Since $\frac{dR}{d\mu }>0$, similar to the stochastic case fast external periodic driving moves the system from a nonlinear regime far from the bifurcation threshold to a linear regime close to to the stability threshold, cf. Fig 3C.

Using the Galerkin approach as in Eq (15), one obtains an analogous expression to Eq (17) for the oscillation frequency but for the periodic forcing case

$$\omega \approx \frac{1}{2\pi \tau }{\text{cos}}^{-1}\left(1/\bar{w}{\tilde{F}}^{\prime }\left[{\bar{u}}_{o}\right]\right)$$ (32)

for small periodic driving amplitudes *I*_0_ and with the fixed point ${\bar{u}}_{o}=\tilde{F}\left[{\bar{u}}_{o}\right]\approx \pi \mu \bar{w}/2\left(\mu \pi -\bar{w}\right)$ for small *μ*. Then after few calculus steps one finds

$$\frac{d\omega }{d\mu }\;\sim -\frac{d\tilde{F}}{d\mu }>0$$ (33)

for small values of *μ*. Hence linearising the response function by fast external stimulation increases the oscillation frequency of the system. Fig 5 shows numerical simulations of Eq (30) for three different driving amplitudes. The oscillation frequency increases with increasing driving amplitude in accordance to the analytical result in Eq (28). This finding resembles the stochastic case for increasing noise level.

To summarize the results above, periodic forcing not only interacts with the dynamics of the network by resonance or entrainment, but also shapes its activity via non-linear effects. This thus implies that the use of weak, high frequency stimulation can mediate frequency transitions in a similar fashion as stochastic inputs.

## Discussion

Previous experimental and theoretical work has shown that gamma-like oscillatory activity is highly dynamic, changing according to stimulus intensity [42]11), spatial features [43,44] and is strongly correlated to the phase of slower frequencies [45,46]. Such gamma oscillations have been shown to build on highly local circuits shaping the timing of interactions between excitatory pyramidal cells and inhibitory interneurons [11]. Slower frequencies, such as alpha activity, have also been shown to be variable. Shifts in the peak alpha frequency have been reliably reported during changes in attentional states [13, 18, 19], during sensorimotor task performance [15], and following intense physical exercise [16].

Alpha oscillations have been shown to engage more spatially extended connections[47], in which propagation delays play an important role [20], suggesting that the mechanisms involved in shaping the peak alpha frequency is different from the one involved for faster, more local frequencies such as gamma for example. Most of the research on synchronous neural dynamics has been devoted to the study of input-induced transitions in- and out of synchronous states, where network firing rate oscillations emerge in presence of strongly correlated drive [30,48,49]. In contrast, we here characterize a smooth approach towards a delayed-induced bifurcation in which the equilibria, stability and peak frequency are impacted by noise or external periodic driving. Using mean-field approaches, we have detailed the stability of the network oscillatory states and derived a frequency tuning curve that relates the frequency of synchronous oscillations to the intensity of the input driving the neurons.

While we have considered constant values of the input intensity in our analysis, results extend to time-varying inputs as well. For instance, the level of noise *D*(*t*) driving neurons would vary according to particular sets of stimuli and\or tasks. In such a case, fluctuations in the noise intensity would be mirrored by concomitant changes in the network peak frequency. What are implications of this dynamic behavior with respect to neural coding? Similarly, experimental setups involving electric periodic stimulation, such as in deep brain stimulation [50,51,52], electric stimulation [53,54].or visual stimulation [55], will induce a shift of frequency [40].

According to the framework we detailed, shifts in the magnitude of inputs to the neurons translate into changes in synchronous oscillations frequencies. Network mean output activity can thus be described by the heuristic relationship

$$u\left(t\right)\approx A\text{sin}\left[2\pi (f_{o}+\Delta \right)t+\phi ]$$ (34)

where *f*_*o*_ is the baseline frequency, Δ is an input-dependent frequency shift, *A* is an amplitude parameter (which may depend on input parameters) and *ϕ* is a random phase. From this perspective, the frequency tuning mechanism can be seen as implementing a frequency modulation (FM) communication scheme coding for input intensity. This hypothesis has been considered on numerous occasions [56,30], and lately gained momentum alongside the development of the “Communication Through Coherence” (CTC) hypothesis in which neural assemblies engage into long-distance communication based on the coherence between oscillatory states [57]. Hence, we hypothesize that the stochastic frequency tuning explored here could potentially implement a gating mechanism allowing the routing of information towards different distal networks, and thus represent an aspect of large-scale neural coding.
